# Knowledge and Attitudes of Small Animal Veterinarians on Antimicrobial Use Practices Impacting the Selection of Antimicrobial Resistance in Dogs and Cats in Illinois, United States: A Spatial Epidemiological Approach

**DOI:** 10.3390/antibiotics12030542

**Published:** 2023-03-08

**Authors:** Setyo Yudhanto, Csaba Varga

**Affiliations:** 1 Department of Pathobiology, University of Illinois Urbana Champaign, Urbana, IL 61802, USA; 2 Carl R. Woese Institute for Genomic Biology, University of Illinois Urbana-Champaign, Urbana, IL 61802, USA

**Keywords:** spatial epidemiology, antimicrobial resistance, antimicrobial use, small animal veterinarian, dogs, cats, Illinois, USA

## Abstract

Inappropriate antimicrobial use in animals and humans has been associated with the emergence of antimicrobial resistance, which has become a global public health concern. Veterinarians’ practice locations and their knowledge and opinions on antimicrobial resistance may influence their antimicrobial prescription practices, which could impact the emergence of antimicrobial-resistant bacteria. This study used a spatial modeling approach to identify areas where veterinarians are knowledgeable about factors that impact the selection of antimicrobial resistance. In addition, we sought to identify regions with higher- and lower-than-expected response rates to our survey to aid future antimicrobial stewardship efforts. A total of 83 veterinarians who treated dogs and/or cats across 34 different Illinois counties responded to our online survey. Most of the responders (90.9%) considered that insufficient doses or duration of antibiotic treatments contribute the most to the selection of antimicrobial resistance. A high proportion of veterinarians (78.7%) attended educational programs on antimicrobial use and resistance; however, only 46.2% were knowledgeable about the current antimicrobial resistance profiles of prevalent bacteria in their area. A mean knowledge score for each county was calculated based on the responses of veterinarians to the survey questions. Local Moran’s I statistic was used to identify counties with high and low knowledge scores. A high knowledge score area in the northeast region and a low knowledge score area in the southeast of Illinois were identified. Using scan statistics with a Poisson model that accounted for the estimated number of veterinarians in a county, a higher-than-expected response rate area was identified in central-east Illinois and a lower-than-expected area in the northeast. This study showed the effectiveness of using geographic analysis and spatial statistics to identify locations where future antimicrobial stewardship programs should focus.

## 1. Introduction

In the past few decades, the emergence of antimicrobial resistance (AMR) has been a concern for human, animal, and environmental health [1,2]. The interaction between animals, humans, and their surrounding environment induces the complexity of the emergence and transmission of AMR and creates a substantial public health challenge [3]. In 2019 alone, infections with antimicrobial-resistant bacterial pathogens caused at least 1.27 million deaths and were associated with an additional 5 million deaths worldwide [4]. In the United States of America (US), the Centers for Disease Control and Prevention estimated that annually 2.8 million infections are caused by antimicrobial-resistant bacteria [5]. It has been shown that the widespread and inappropriate use and overuse of antimicrobials have the highest influence on the selection of AMR in humans, animals, and their environment [6,7,8].

Small animals could be considered reservoirs of antimicrobial-resistant bacteria posing a zoonotic transmission risk as they live near humans and are frequently treated with antimicrobials [9,10]. One of the challenges in limiting the spread of AMR is the awareness and knowledge of small animal veterinarians related to factors that influence the selection of antimicrobial-resistant bacteria [11,12]. In the small animal hospital setting, besides the inappropriate use of antimicrobials, other factors were identified to be associated with the emergence of AMR, including inadequate infection control and prevention practices (i.e., deficient disinfection and sterilization of instruments) [13]. Inappropriate infection prevention measures in veterinary hospitals could also facilitate the spread of antimicrobial-resistant bacteria among animals [3], between animals and their owners and/or healthcare staff [9]. These problems can limit the effectiveness of preventing and managing antimicrobial-resistant bacterial infections [14]. However, the healthcare staff may not fully understand the implications of the antimicrobial prescribing practices and infection control measures’ contribution to the selection of AMR [15]. Understanding small animal veterinarians’ knowledge and attitude toward antimicrobial use (AMU) and AMR is important to help veterinary stakeholders in designing effective antimicrobial stewardship programs by developing guidelines and recommendations for judicious antimicrobial use [14,16].

Spatial epidemiology methods have been applied successfully to identify higher-than-expected area-level infection rates in humans [17,18], pets [19], livestock [20], and wildlife [21,22]. To better understand and assess geographical variations in small animal veterinarians’ knowledge and attitudes towards antimicrobial use and infection prevention practices that impact the selection of AMR spatial epidemiology methods might be useful because these factors might be influenced by a variety of local factors, including the availability of antimicrobial drugs, guidelines on antimicrobial use and infection prevention, and the prevalence of bacterial pathogens [23]. In addition, geographical variations in antimicrobial use practices might lead to a variation in the distribution of antimicrobial-resistant pathogens [24,25,26].

To the best of our knowledge, no previous study assessed issues related to antimicrobial stewardship in Illinois, and to address this knowledge gap, our study aims to assess Illinois small animal veterinarians’ knowledge and attitudes related to antimicrobial stewardship that impacts the selection of AMR and investigates whether these factors vary by location. In addition, we aim to identify areas where small animal veterinarians willingly participated in our study and regions where additional antimicrobial stewardship outreach programs are needed.

## 2. Results

### 2.1. Demographic Data

The demographic characteristics of the small animal veterinarians who participated in the study are presented in Table 1. Of the total 83 responders included in the analysis, 66.3% were females, and 31.3% were males. Almost half of the responders (48.8%) were 50 years or older. Most responders worked at a veterinary primary care hospital/clinic (74.7%), while 14.5% worked at a veterinary academic teaching hospital and 7.2% at veterinary emergency facilities. Most of the responders were exclusively treating small animals (75.9%). Almost half of the responders (43.4%) were the owners of the clinic/hospital, and they commonly had 1–2 veterinarians on staff (36.6%). The majority of responders (77.1%) had Doctor of Veterinary Medicine (DVM) degrees, while the remaining veterinarians had additional post-DVM degrees or certifications. Most of the responders (73.5%) had more than 11 years of experience prescribing antimicrobials to dogs and/or cats.

### 2.2. Awareness of Antimicrobial Resistance and Antimicrobial Use

The responders stated that they were familiar with the AMR topic (43.6% were very familiar, and 46.2% were moderately familiar); 78.7% reported that they attended educational programs on antimicrobial use (AMU) and AMR after graduation, and 46.2% were aware of the current AMR profiles of prevalent bacteria in their area. Furthermore, the responders indicated that they were concerned about infections with antibiotic-resistant pathogens when treating their patients (53.8% moderately concerned, and 33.3% very concerned). Meanwhile, just over a quarter (26.3%) of the responders stated that they have antimicrobial prescription guidelines at their facility. Before starting antimicrobial treatment, 45.5% of the responders described that they sometimes performed cytology before treating suspected bacterial infections, while 10.4% always and 36.4% often performed cytology (Table 2).

### 2.3. Opinion of Illinois Small Animal Veterinarians on Antimicrobial Use and Infection Prevention Practices’ Contribution to the Selection of Antimicrobial Resistance

When being asked about the likelihood of certain antimicrobial use practices’ effect on the selection of antimicrobial-resistant bacteria (Table 3), most of the responders (58.4% extremely likely, and 32.5% likely) believed that inadequate dose or duration of antibiotic treatments contribute to the selection of AMR, followed by prescription of broad-spectrum antibiotics (19.2% extremely likely, and 53.8% likely), and empirical antibiotic therapy without performing culture and susceptibility tests (18% extremely likely, and 55% likely). Moreover, only above half (16.7% extremely likely, and 35.9% likely) and around a quarter (9% extremely likely, and 18% likely) of the responders stated that improper infection prevention and prescription of antibiotics extra-label contributed to the emergence of AMR, respectively.

### 2.4. Cluster Analysis of Factors Impacting the Selection of Antimicrobial Resistance

**Figure 1:**
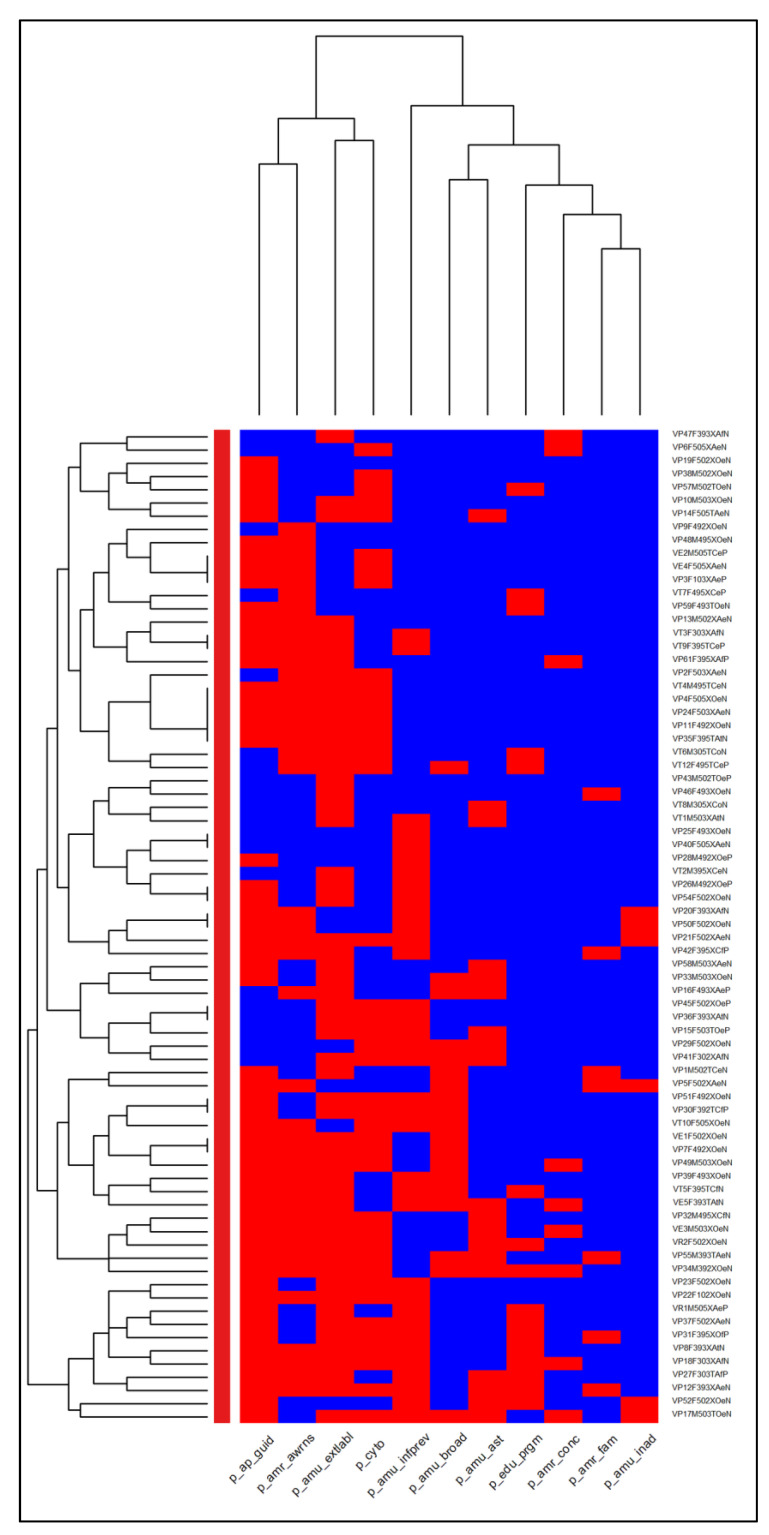
Hierarchical clustering dendrogram of small animal veterinarians’ awareness of AMR and belief of practices that contribute to the selection of antimicrobial resistance. Red color, no (0); blue color, yes (1). p_ap_guid: antimicrobial prescription guideline in the practice facility; p_amr_awrns: awareness of local antimicrobial resistance profile; p_amu_extlabl: prescription of antibiotics extra-label; p_cyto: perform cytology before treating bacterial infection; p_amu_infprev: improper infection prevention in the facilities; p_amu_broad: prescription of broad-spectrum antibiotics; p_amu_ast: empirical treatment without antimicrobial susceptibility tests; p_edu_prgm: attending educational programs on AMU and AMR after graduation; p_amr_conc: concerned about AMR bacteria; p_amr_fam: familiar with AMR topic; p_amu_inad: inadequate dose/duration of antibiotics treatments.

A cluster analysis of small animal veterinarians’ attitudes toward factors impacting the selection of AMR is shown in Figure 1. The clustering dendrogram (heatmap) evaluated the clustering of opinions on various factors that impact the selection of AMR (columns) of the survey responders (rows). In the columns, two main clusters of opinions were identified. The first cluster located on the right side of the heatmap columns included responses about familiarity with the topic of AMR, concerns with AMR when treating patients, attendance of educational programs on AMU and AMR, and beliefs that several AMU practices were likely to contribute to AMR (i.e., prescription of broad-spectrum antibiotics, empirical antibiotic therapy without performing culture and susceptibility tests, and inadequate dose or duration of antibiotic treatments). The second cluster located at the left side of the heatmap columns included responses that reported the practice facility does not have antimicrobial prescription guidelines, were not aware of the current local AMR profiles, does not believe prescribing antibiotics extra-label contributes to AMR, and are less likely to perform cytology before treating suspected bacterial infections. In the rows of the heatmaps, two clusters of responders were observed. The first cluster was at the bottom of the heatmaps where the veterinarians were mostly familiar with the AMR topic and were concerned about the selection of AMR when treating their patients and believed that inadequate dose/duration of antibiotics treatments, the prescription of broad-spectrum antibiotics, and empirical treatment without antimicrobial susceptibility tests were likely to contribute to the selection of AMR. However, in this cluster, the responders reported that they do not have antimicrobial prescription guidelines at their practice facility, were not aware of the current AMR profiles of prevalent bacteria in their area, were less likely to perform cytology before treatment initiation, did not attend the educational programs on AMU and AMR, and believed that prescription of antibiotics extra-label and improper infection prevention in veterinary facilities were less likely to contribute to the selection of AMR. The second cluster included several sub clusters with similar opinions from different demographic characteristics of responders.

### 2.5. Spatial Analysis of Response Rates and Knowledge Scores of Illinois Small Animal Veterinarians

#### 2.5.1. Mapping of the Survey Response Rates

**Figure 2:**
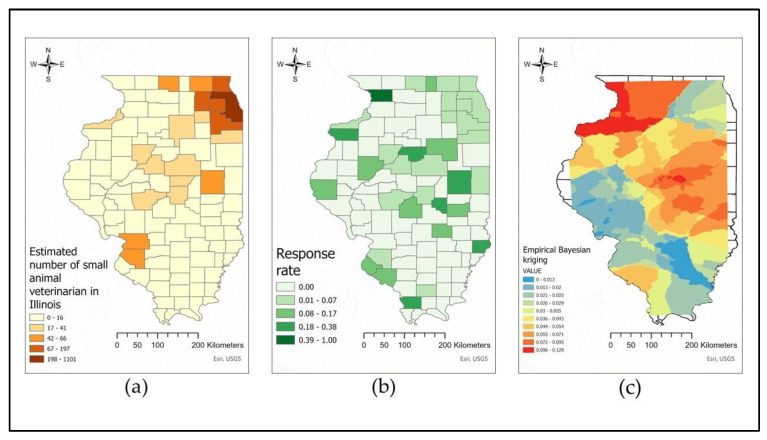
Choropleth map of the estimated number of small animal veterinarians in Illinois (**a**). Choropleth map of response rates of the survey on AMR and AMU practices among Illinois companion animal veterinarians (**b**). Isopleth map of the spatial interpolation of survey response rates by using empirical Bayesian kriging (**c**).

A total of 83 responders from 34 different counties were included in the response rate analysis (Table S1; Figure S1). We estimated the county-level number of small animal veterinarians in Illinois considering the number of households in a county, the estimated number of veterinarians per household, and the proportion of small animal veterinarians. Estimated county-level numbers of small animal veterinarians ranged from 1 to 1101. Counties with high small animal veterinarian numbers were identified in the northeast, central, and southwest of Illinois (Figure 2a). Based on the county-level survey responses of small animal veterinarians and accounting for the estimated number of small animal veterinarians in each county, we calculated the survey response rate (mean of 3 responses per 100 small animal veterinarians), which is shown in Figure 2b. The highest response rate was from Carroll County (response rate = 1), followed by Woodford County (response rate = 0.38). The responders’ practice location was from 34 different counties in the state of Illinois, with the highest number of responses coming from the counties of Champaign (*n* = 13, 15.7% total responders), followed by Cook (*n* = 9, 10.8%), DuPage (*n* = 6, 7.2%), Lake (*n* = 6, 7.2%), and Will (*n* = 6, 7.2%) (Table S1; Figure S1). The empirical Bayesian kriging of the survey response rates (Figure 2c) illustrates several regions with a high response rate, including northwest and east-central Illinois, with values ranging from 0 to 0.129.

#### 2.5.2. Global Spatial Cluster Analysis of Survey Response Rates

The Incremental Spatial Autocorrelation (Global Moran’s I) Tool assessed the global clustering of response rates by searching for a series of increasing distances and assessing the intensity of global spatial clustering at each distance; however, the analysis did not identify a distance with statistically significant high value (e.g., peak) (Figure S2). Therefore, the “contiguity edges corners” conceptualization parameter was used for the local spatial cluster analysis of response rates.

#### 2.5.3. Local Spatial Cluster Analysis of Survey Response Rates

**Figure 3:**
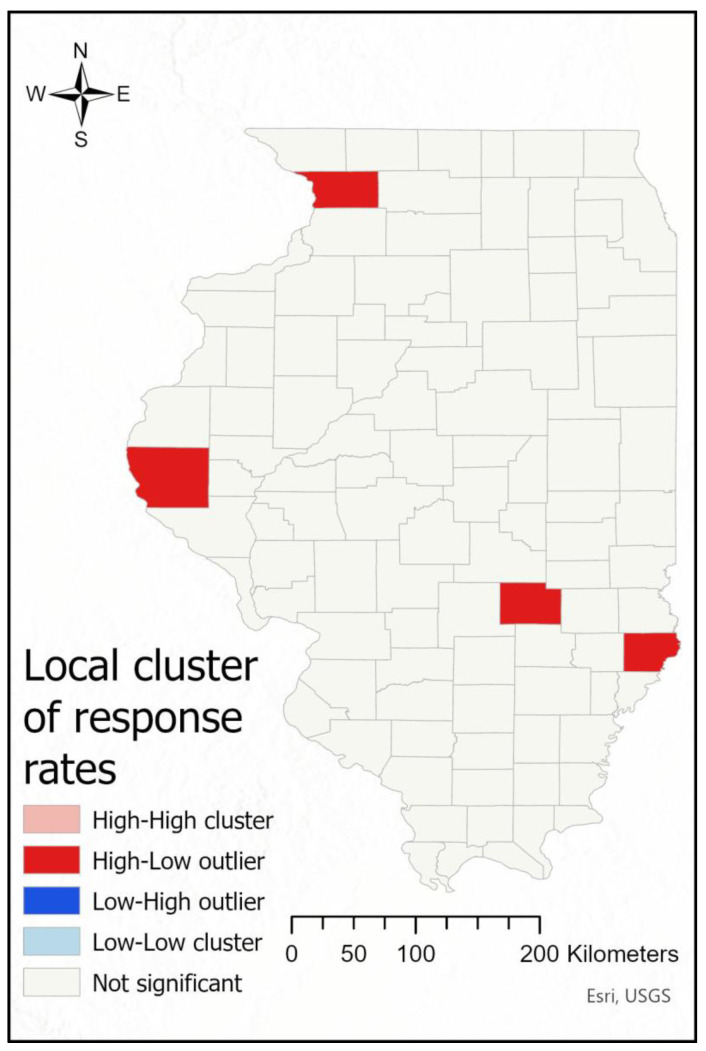
Cluster and outlier analysis (local Moran’s I) results of the survey response rates.

The Cluster and Outlier Analysis (Anselin local Moran’s I statistics) Tool detected five counties with high response rates that were surrounded by counties with low response rates (high-low outlier clusters) (Figure 3; Table S2).

#### 2.5.4. Spatial Scan Statistic of Response Rates

**Figure 4:**
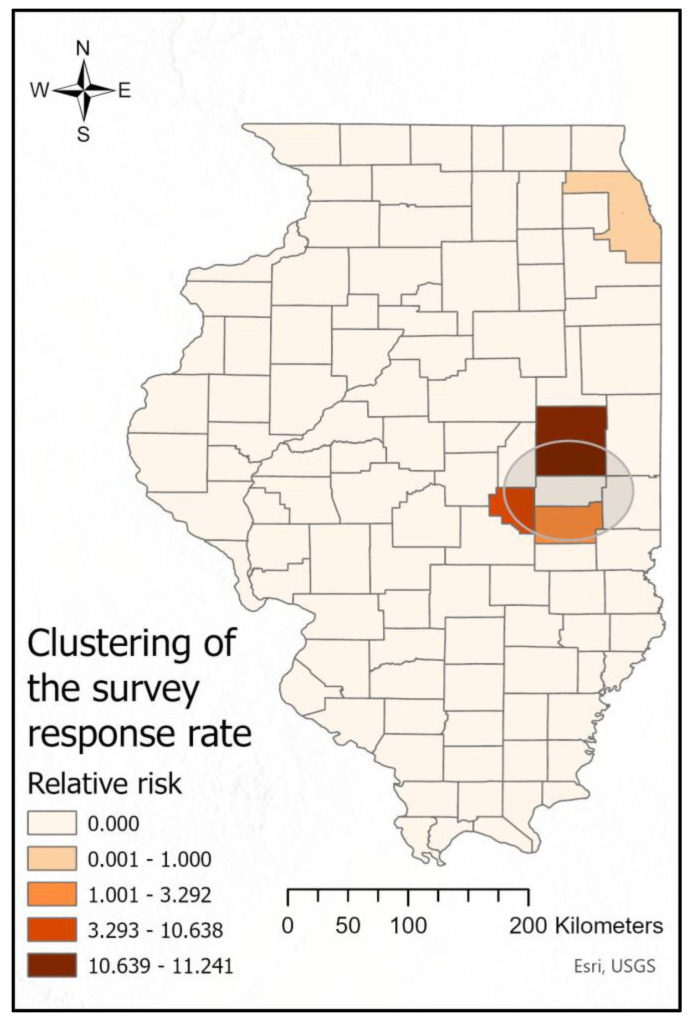
Spatial clusters of response rates of Illinois small animal veterinarians identified by scan statistics using a Poisson model.

The purely spatial scan statistics using a discrete Poisson model, having the relative risk (RR) as the measure of effect, detected two significant (*p*-value < 0.001) local clusters, one with higher-than-expected (RR = 9.52) and one with lower-than-expected (RR = 0.18) response rates (Figure 4). Four counties were included in the higher-than-expected response rate cluster (Douglas, Coles, Moultrie, and Champaign). This cluster included 15 responses, while only 1.88 responses were expected. Cook County was identified as a lower-than-expected response rate spatial cluster with 9 responses, while 33.88 responses were expected (Figure 4). We also calculated and presented the RR for each county included in the significant spatial cluster that showed the highest RR for Champaign County and the lowest for Douglas County (Figure 4).

#### 2.5.5. Mapping of Knowledge Scores

**Figure 5:**
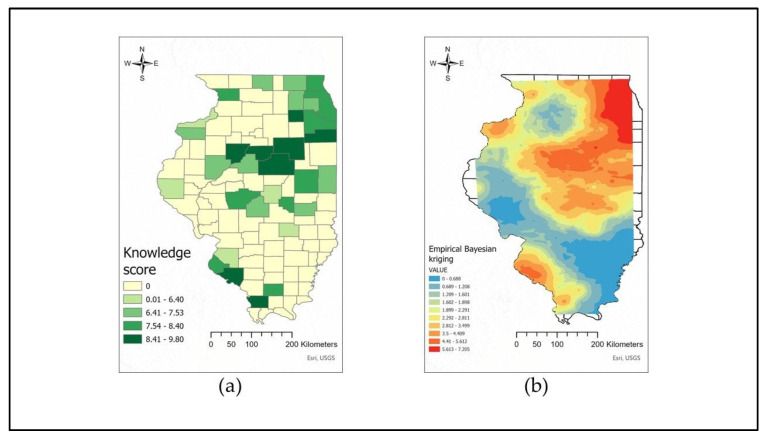
Choropleth map of the survey knowledge scores (**a**). Isopleth map of knowledge scores applying spatial interpolation by using empirical Bayesian kriging (**b**).

Of the 83 responses, 75 responses were included in the knowledge score calculation. The average knowledge score by county ranged from 0 to 9.8, which is illustrated in Figure 5a. Kankakee County showed the highest knowledge score (9.8 out of max. 11), followed by Woodford (9.4) and Union County (9.4). The empirical Bayesian kriging of the knowledge scores is presented in Figure 5b. The values ranged from 0 to 0.72 and displayed regions with high knowledge scores in the northeast, northwest, central, and southwest regions of Illinois.

#### 2.5.6. Global Spatial Cluster Analysis of Knowledge Scores

**Figure 6:**
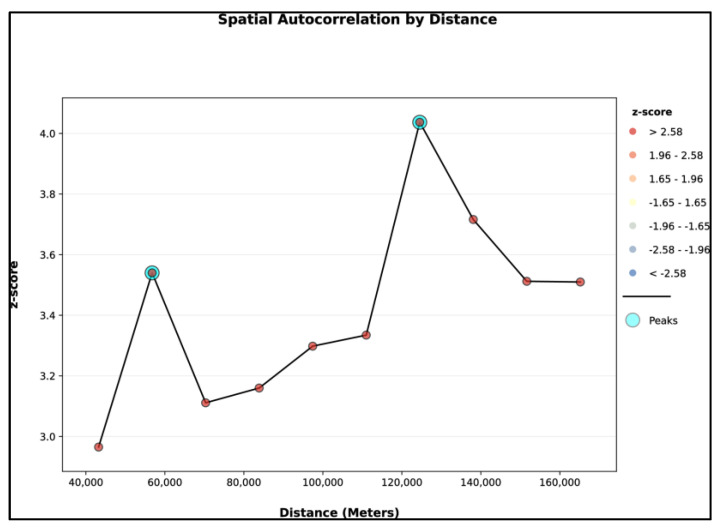
Incremental spatial autocorrelation (Global Moran’s I statistics) analysis results of the knowledge score.

The Incremental Spatial Analysis (Global Moran’s I statistics) Tool results are shown in Figure 6. The first significant knowledge score peak was identified at 56.77 km, while the maximum knowledge score peak (highest Z-score) was identified at 124.53 km. Thus, the 124.53 km distance band and the “zone of indifference” conceptualization parameter were used for the local spatial cluster analysis. 

#### 2.5.7. Local Spatial Cluster Analysis of Knowledge Scores

**Figure 7:**
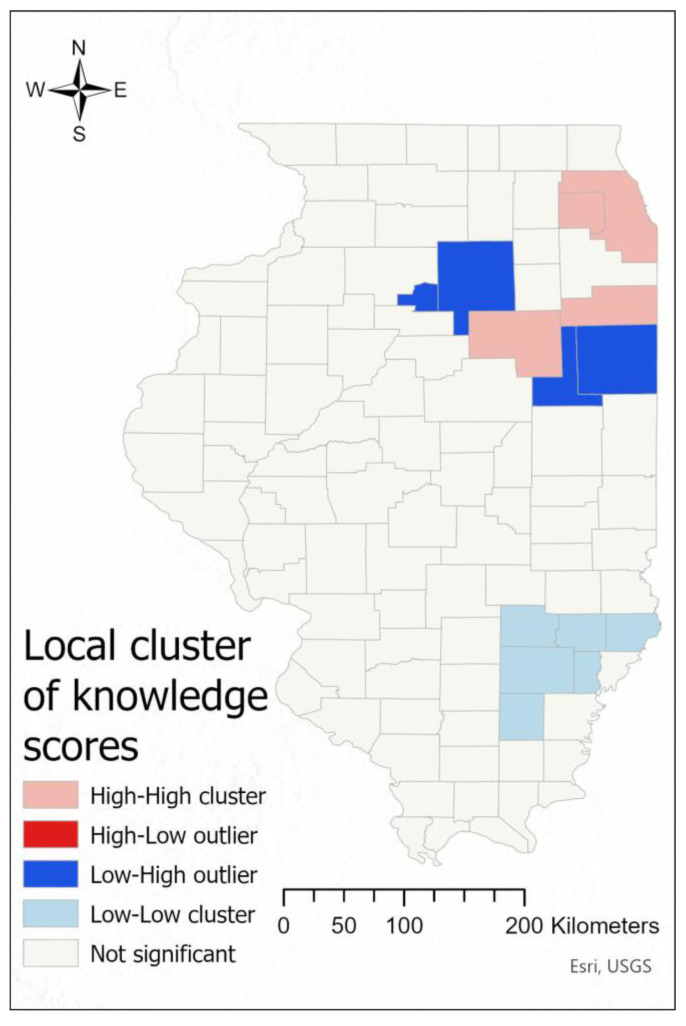
Cluster and outlier (local Moran’s I) analysis result of survey knowledge scores.

The Cluster and Outlier Analysis (Anselin local Moran’s I) Tool results of the knowledge scores are illustrated in Figure 7. Four counties were identified with high knowledge scores that were surrounded by counties with high knowledge scores (high-high cluster), four counties with low knowledge scores were surrounded by counties with high knowledge scores (low-high outlier cluster), and six counties were identified that had low knowledge scores and were surrounded by counties with low knowledge scores (low-low cluster) (Figure 7; Table S2).

## 3. Discussion

This research study evaluated the knowledge, attitudes, and practices of small animal veterinarians in Illinois related to antimicrobial stewardship that were obtained through an online survey. Spatial epidemiology approaches were used to investigate geographical variations in small animal veterinarians’ knowledge and attitudes towards AMU and AMR and to assess their opinions on the impact of AMU and infection prevention practices on the selection of AMR. In addition, to guide future antimicrobial stewardship outreach efforts, we identified areas with high and low response rates.

The results of this hypothesis-generating study showed an awareness of AMR among Illinois small animal veterinarians, where the majority of veterinarians were concerned about AMR when treating their patients. Even though many studies in the United States and globally have focused on veterinarians’ knowledge and perceptions regarding AMU and AMR [11,16,27,28,29,30], few studies have used spatial analytical approaches to study veterinary care issues [31] in particular observing the geographical difference of antimicrobial stewardship that impacts the selection of AMR, which may lead to local variations in AMR patterns of prevalent bacteria [24,25,27].

The emergence of AMR in the USA and worldwide urged animal health stakeholders to develop stewardship programs to reduce the improper use of antimicrobials [32,33]. Previous studies emphasized the importance of veterinarians being familiar with the topic of AMR and the benefit of professional continuing education programs to improve antimicrobial stewardship in preventing and addressing AMR [28,34]. In this study, most of the veterinarians (78.7%) attended educational programs on AMU and AMR post-graduation. However, we found that less than half of our survey responders were aware of the local AMR patterns of bacteria (46.2%), and only 26.3% of the veterinary facilities had antimicrobial prescription guidelines. Furthermore, only 10.4% of the veterinarians stated that they always performed cytology for suspected bacterial infections before initiating treatments (36.4% reported often, and 45.5% stated they sometimes perform cytology). Performing cytology before starting antimicrobial treatment of certain bacterial infections was described by previous studies as an important tool in limiting the selection of AMR [35,36,37].

In this study, by using a clustering dendrogram (heatmap) for the responses of Illinois small animal veterinarians’ on AMR and AMU awareness and their opinions on the different AMU practices impacting the emergence of AMR, we could identify commonalities (clusters) that need attention when antimicrobial stewardship programs are developed. In particular, a concerning cluster of responders was identified who did not have antimicrobial prescription guidelines at their practice facility, did not know the current AMR patterns of bacteria in their area, did not perform routine cytology before initiating treatment, and did not attend educational programs on AMU and AMR. This finding suggests that there is a need to build strategies aimed at increasing antimicrobial stewardship among Illinois small animal veterinarians to help promote judicious AMU practices. One of the approaches that could be used is to identify geographical differences in small animal veterinarians’ AMU practices and their knowledge of factors that affect the emergence of AMR.

### Spatial Epidemiological Approach to Assess Response Rates and Knowledge Scores of Illinois Small Animal Veterinarians

Our study used a stepwise exploratory spatial analysis to evaluate the geographical variations in small animal veterinarians’ knowledge and attitudes towards AMU that impact the selection of AMR and to identify areas with high and low response rates. In the first step, we used mapping by constructing choropleth and isopleth maps to assess visually the distribution of knowledge scores and response rates. Next global cluster analysis was used to assess the extent of clustering across the study area and to identify a distance with the highest statistically significant global clustering. In the last step, we applied local clustering analysis to identify areas with high, low, or outlier knowledge scores and response rates. These areas should be followed up to understand local factors affecting antimicrobial stewardship.

#### 3.1.1. Assessing the Response Rates of Illinois Small Animal Veterinarians

In the present study, we used an online survey method, which was described by several previous studies to have advantages even though the response rate is lower compared to the other survey methods due to the lack of in-person engagement between the researchers and the participants [38,39,40]. To address this issue, we applied strategies to increase the response rate of online surveys suggested by previous studies [39,41,42]. Our survey was distributed via individual emails to the target population (i.e., small animal veterinarians) through the Illinois State Veterinary Medical Association (ISVMA), and two email reminders were also sent. Additionally, a cover letter was included at the beginning of the survey explaining that participation in the survey will be anonymous and that the collected data will be handled in compliance with privacy regulations. In addition, an advertisement with a link to the survey was distributed through the ISVMA e-newsletter. To increase the likelihood of completion, we designed the survey to be completed in approximately 10 min. Our response rate was 3 per 100 small animal veterinarians, which was similar to previous studies from the USA and Australia [11,28]. In addition, prior studies evaluating online survey response rates showed that a response rate of less than 11% was not uncommon for online surveys [42,43].

When evaluating the response rates across Illinois, we used a novel method by first calculating for each county the estimated number of small animal veterinarians based on a computation described by a previous study that estimated one veterinarian per 1500 households [44]. Next, we visualized the estimated number of small animal veterinarians in Illinois on a choropleth map and also constructed a choropleth map that illustrated the response rate (dividing the number of responses in a county by the estimated small animal veterinarians in that county) for each county. This method helped us to understand the distribution of the overall spatial response rate and visually identify the counties with high and low response rates. We found that the response rates of our survey followed the estimated number of veterinarians in each county except for the northeast region of Illinois, where the response rate was low, even though we received nine responses. This region is the highest populated area in Illinois, consisting of the city of Chicago, where the highest estimated number of small animal veterinarians work. In addition to the choropleth map, we made an isopleth map by using spatial interpolation and applying the spatial empirical Bayesian kriging method, which removed the strict boundaries of counties, identified hot spot (high response rates) and cold spot (low response rates) areas, and was easier to evaluate than choropleth maps.

To identify areas with significantly higher- or lower-than-expected response rates, we used retrospective scan statistics with a discrete Poisson model that accounted for the estimated number of veterinarians in each county. The higher-than-expected response rate cluster was observed in Champaign County and three neighboring counties (Douglas, Coles, and Moultrie), which may be due to the location of the College of Veterinary Medicine, University of Illinois in Champaign County, where there might be a higher willingness to participate in research studies among veterinarians working in an academic setting and among small animal veterinarians employed in primary veterinary practices. The low response rate area in northeast Illinois identified visually in the choropleth map was confirmed statistically by the purely spatial scan statistics. 

The cluster-outlier analysis of response rates identified several areas where future studies should be conducted. Our findings on the distribution and clustering of response rates can also assist health authorities when delivering antimicrobial stewardship programs to hinder the emergence of AMR.

#### 3.1.2. Knowledge Scores of Illinois Small Animal Veterinarians

In our study, we developed a knowledge score system based on the survey responses of veterinarians and calculated an average score for each Illinois county. We then presented the distribution of knowledge scores in choropleth maps to visually identify counties with high and low knowledge scores. The limitation of this approach was that areas with no responses received a knowledge score of zero. To address this issue, we constructed an isopleth map by spatially interpolating the knowledge scores using the empirical Bayesian kriging method. The assumption was that the veterinarians whose practices are in the same area or nearby areas would have comparable knowledge scores. Another advantage of spatial interpolation is that it omits the county boundaries and utilizes the nearest observations knowledge scores to estimate scores at locations with limited information [45]. On the isopleth map, a high knowledge score area (hot spot) could be seen in the northeast of Illinois, which was later identified by the local cluster analysis as a statistically significant high-high knowledge score cluster. Two outlier knowledge score (low-high cluster) areas where a low knowledge score county is surrounded by high knowledge score counties were identified, one in the northeast of Illinois and a second in Champaign County. Interestingly, when compared to the response rate maps, the northeast of Illinois, which had a low response rate, had been identified as a high knowledge score cluster. These results are encouraging because they show a high awareness and understanding of the issues of AMR and AMU in the area with the highest estimated number of veterinarians. Whereas the counties in the southeast of Illinois that were identified as low-low knowledge score clusters in the Cluster and Outlier (Anselin local Moran’s I) analysis had a low response rate and a low estimated number of veterinarians, thus must be interpreted with caution. The findings of this analysis could help in the development of a targeted stewardship program to improve AMU practices among outlier areas and provide insight for further studies in the low cluster areas.

In the context of antimicrobial stewardship among small animal veterinarians, spatial epidemiology could be used to study the patterns of antimicrobial use in different geographical areas and how these patterns may impact the spread of AMR among local small animal populations. Furthermore, access to educational opportunities might differ among areas that might impact antimicrobial stewardship among veterinarians. Studying these variations can help identify areas where judicious antimicrobial use is practiced or lacking and can help animal health stakeholders to improve antimicrobial stewardship programs in these areas. The geographical variations in knowledge, attitudes, and practices of small animal veterinarians highlight the need for careful monitoring of these practices to ensure that they are both effective and sustainable in the long term.

## 4. Study Limitations

A few limitations should be considered while interpreting our study. This survey used an online voluntary methodology which provided the potential responders with a non-personalized web link to maintain anonymity. This study should be interpreted with caution and might not represent all small animal veterinarians in Illinois due to a non-response bias. In addition, not all counties in Illinois had responders, the background population was based on estimation, and veterinarians working in academic settings were overrepresented. While recognizing the challenges in gathering information from every Illinois county, we used spatial interpolation to generate estimates for areas with no responses. Nevertheless, our spatial epidemiology method established a new way of approaching the factors affecting the emergence of AMR. Lastly, when interpreting our results, one should consider that the knowledge score was a composite overall score calculated from responses of all veterinarians in a county, and the responses to questions do not indicate individual veterinarians’ knowledge about AMR and antibiotic stewardship, which might be influenced by veterinarians’ demographics, years in practice, and practice type.

## 5. Materials and Methods

### 5.1. Research Framework 

#### Survey Development

**Figure 8:**
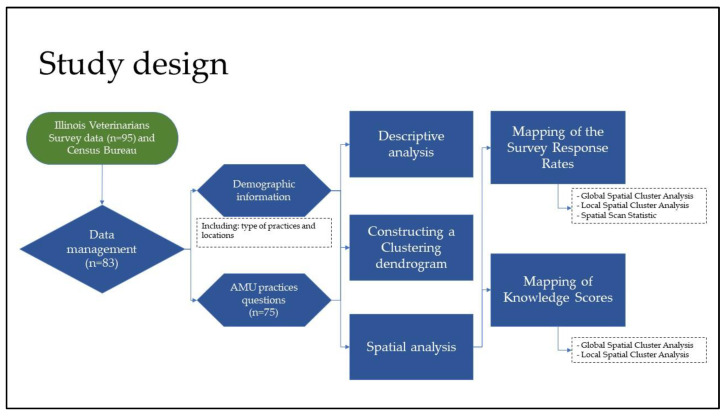
Spatial epidemiological approaches to evaluate spatial clustering of knowledge on AMU and AMR and response rates of small animal veterinarians in Illinois, USA.

This survey was developed using the Qualtrics^XM^®, Version March 2023 (Qualtrics, Provo, UT, USA) software, a web-based tool that could be completed only by licensed veterinarians who were prescribing antimicrobials to dogs and/or cats in Illinois, USA (Figure 8). First, we collected and adapted questions from previously published studies [11,16,28] and developed a draft questionnaire to obtain information on Illinois small animal veterinarians’ knowledge and attitude toward AMU and factors that impact the selection of AMR. The questionnaire contained 19 questions, which were divided into three sections asking Illinois small animal veterinarians about their: (1) demographic information, (2) awareness of AMR and antimicrobial stewardship, and (3) opinion on the likelihood of certain antimicrobial use practices’ contribution to the selection of AMR. The questionnaire was designed for the study participant to be completed in around 10 min and contained multiple-choice (*n* = 8), yes/no (*n* = 3), and 5-point Likert scale (*n* = 8) questions.

The pretesting of the survey was conducted in two steps. First, the draft questionnaire was sent to 4 experts working in small animal medicine, clinical pharmacology, and microbiology at the University of Illinois Urbana-Champaign, College of Veterinary Medicine (CVM) to review the relevance of questions related to the study objectives and to identify potential ambiguous questions. After addressing their suggestions, next, the questionnaire was sent to graduate students at the Department of Pathobiology, CVM, who hold a DVM degree to assess its clarity and identify confusing or misleading questions.

### 5.2. Ethics, Participation Consent, and Survey Administration

This study was approved and deemed exempt under 45 CFR 46 regulations by the University of Illinois Urbana-Champaign Office for the Protection of Research Subjects (OPRS) (no. 23274). Each survey participant had to consent to participate voluntarily at the beginning of the anonymous online survey, and no incentive was given to participants.

The online survey was administered to small animal veterinarians through the Illinois State Veterinary Medical Association (ISVMA) members’ (approximately 3000 members) mailing list. The members of the ISVMA were requested to participate anonymously in this study via a non-personalized Uniform Resource Locator (URL) link provided. The survey period was between 7 September and 7 November 2022. During the study period, one initial email and two reminder emails were sent to ISVMA members, and two advertisements were published in the ISVMA e-newsletter.

### 5.3. Data and Spatial Analysis

#### 5.3.1. Descriptive Analysis and Clustering Dendrogram

All descriptive analyses were performed in R version 4.1.2 using RStudio-2022.07.1–554. The demographic variables included in the analysis were: gender, age, type of veterinary practice facility, type of veterinary service, number of veterinarians, and their role at the veterinary facility and the completion of additional post-DVM degrees or certifications. The variables distribution and their 95% confidence intervals were computed for demographic information and categorical variables (i.e., Yes/No, multiple choice, and Likert scale). The responses to “Yes/No” questions were recoded as 1/0; meanwhile, for the 5-point Likert scale, the answers were given a point scale from 1 to 5. 

Additionally, the R software heatmap.2 package combined with RColorBrewer and ggplots libraries were used to assess the knowledge score patterns in terms of their similarity in the responders’ characteristics by constructing a clustering dendrogram and illustrating it in a heatmap. The heatmap included 11 variables representing small animal veterinarians’ attitudes toward factors impacting the emergence of AMR (Table 2 and Table 3). This heatmap illustrated a dendrogram for the result of a hierarchical single-linkage clustering method with Ward’s hierarchical clustering method using Euclidean distances [46]. For the heatmap, the Likert scale variables were recoded as 1 (extremely or likely, very familiar or moderately familiar, very concerned or moderately concerned, always or often) and 0 (the other scales).

#### 5.3.2. Survey Response Rate 

The sample size needed in the study was estimated to be at least 67 responders, assuming that 50% of small animal veterinarians answer a question identically, with a 90% confidence level, a 10% margin of error, and an estimated population size of 2697 (the estimated number of ISVMA members who treat small animals).

A total of 95 participants responded to the survey. Of the total, 83 responders who completed the demographic section were included for further analysis (Table S1). The response rate (RR) was calculated based on the estimated number of veterinarians across the USA provided by a previous study [44], which estimated that there was approximately one veterinarian per 1500 households (Figure 4a). This approximation was used to estimate the number of veterinarians treating small animals in Illinois by accounting for the proportion of veterinarians who treat dogs and cats in Illinois provided by a previous survey completed by ISVMA among its members that described that 69% were small animal veterinarians and 14% were mixed animal veterinarians. Accordingly, our survey’s response rate for each county was then calculated by dividing the number of responses by the estimated number of veterinarians who treat dogs and/or cats in Illinois.

$$\begin{array}{l} Response\;Rate\\ =\frac{Number\;of\;responses}{Estimated\;number\;of\;all\;veterinarians\;in\;Illinois\;\times \;percentage\;of\;Illinois\;veterinarian\;who\;treat\;dogs\;and\;cats\;} \end{array}$$

#### 5.3.3. Knowledge Score

The knowledge score was determined by summation of responses in 2 sections of the survey (i.e., 11 variables): (1) antimicrobial resistance awareness and antimicrobial use practices among small animal veterinarians in Illinois (Table 2); and (2) the likelihood of several antimicrobial use practices’ contributions to the selection of AMR (Table 3). Only the responders who completed all questions in these two sections were included in this analysis. For each “Yes/No” question, the variables were given the score “1” for “Yes” and “0” for “No”; as for the 5-point Likert scale, after the given score 1–5, the points were normalized by dividing the score by 5. The knowledge score represented the sum of the normalized Likert scale scores and the “Yes/No” scores of the 11 variables.

### 5.4. Spatial Analysis

#### 5.4.1. Study Area and Data Sources

The state of Illinois is in the Midwest region of the US, with a latitude range of 42.5° N to 37° N and a longitude range of 87.5° W to 91.5° W. The area of Illinois is approximately 150,000 km^2^ and consists of 102 counties (Figure S1). Additional information to estimate the number of veterinarians in Illinois was obtained from the US Census Bureau. Data on the demographic information were extracted for 2022, including the population in Illinois per county and the number of households per county. All data were aggregated at the county level and subsequently used in the county-level analyses. The shapefiles of Illinois, including the boundaries of each county to generate the maps, were also obtained from the US Census Bureau. The response rates were estimated based on the American Veterinary Medical Association (AVMA) census of veterinarians conducted in 2019 [44] and the ISVMA survey of its members in 2019. For all spatial analysis, the maps were projected to NAD 1983 UTM Zone 16N.

#### 5.4.2. Map Development for Response Rates and Knowledge Scores

The maps for this study were created using ArcGIS Pro version 3.0.3 (Environmental Systems Research Institute, Inc. (ESRI), Redlands, CA, USA). A manual interval classification scheme was used for the choropleth map presenting the geographic distributions of the estimated number of the study population, and Natural Jenks classification was used to classify the intervals for the response rate and knowledge score choropleth maps [47].

The empirical Bayesian kriging method was conducted to observe the county-level spatial interpolation of response rates and knowledge scores. The spatial dependence of these variables across Illinois counties was measured using a semivariogram function that represents the distance and direction of measured locations (i.e., counties). The empirical Bayesian kriging used a restricted maximum likelihood estimation and developed several semivariograms to account for the error when estimating the semivariogram [48,49].

#### 5.4.3. Global Spatial Cluster Analysis of Response Rates

Incremental Spatial Autocorrelation (Global Moran’s I) Tool was used to assess the global clustering of response rates for ten distance bands. Measurement of the global spatial autocorrelation was based on the locations (i.e., counties centroids) and the values (i.e., response rates for each county) of the features simultaneously. The starting distance of the incremental spatial autocorrelation analysis was represented by the distance at which each county had at least one neighbor. The analysis evaluated whether the response rate was spatially clustered or randomly dispersed with the null hypothesis of complete spatial randomness of area-level response rates. In addition, Moran’s I Index value and a z-score and p-value were also calculated for each distance increment. The analysis of the global clustering of response rates did not detect significant clustering (Figure S2). Thus the “contiguity edges corners” conceptualization parameter was used for the local spatial cluster analysis [50,51].

#### 5.4.4. Local Spatial Cluster Analysis of Response Rates

The evaluation of the cluster analysis for local indicators of spatial autocorrelation of response rates was performed using the Cluster and Outlier (Anselin local Moran’s I) Tool [47,48]. In the analysis, each county was represented by a polygon and its response rate. Distances were measured by the Euclidean distance method, which measures a straight-line distance between the centroid of two polygons. The null hypothesis assumed spatial randomness of counties with high or low rates. The analysis provided a z-score and p-value that were associated with the normal distribution. A statistically significant p-value (*p* ≤ 0.05) with a high (+) or low (−) z-score (± 1.96) in the tails of the normal distribution curve signified a significant cluster. The analysis identified local response rates, represented as a high-high cluster, a low-low cluster, and spatial outliers (high-low and low-high). A positive Moran’s I value indicated that the area (i.e., county) is surrounded by areas that have similar rates (a high-rate county surrounded by high-rate counties (high-high cluster) and a low-rate county surrounded by low-rate counties (low-low cluster). A negative Moran’s I value indicated that a county was surrounded by counties with dissimilar rates (high-low, low-high clusters).

#### 5.4.5. Spatial Scan Statistic of Response Rates

A retrospective scan statistic was performed using the SatScan^TM^ v10.1 software to identify spatial clusters of response rates. Purely spatial scan statistic with a discrete Poisson model was used in the analysis, which assumes that the response rates are Poisson-distributed, based on the number of small animal veterinarians in Illinois as the background population [52]. High- and low-rate clusters were investigated using a 50% circular scanning window. The scanning window scanned across the counties and simultaneously compared the expected and observed rates inside the scanning windows to the outside, using 999 replications of Monte Carlo hypothesis testing [53]. A high-rate cluster (i.e., RR > 1) was identified when the observed rate was significantly (*p*-value ≤ 0.05) higher than expected inside the scanning window compared to the outside. A low-rate cluster (i.e., RR < 1), on the other hand, defined areas with lower-than-expected rates inside the scanning window compared to the outside.

#### 5.4.6. Global Spatial Cluster Analysis of Knowledge Scores

The Incremental Spatial Autocorrelation (Global Moran’s I) Tool was used to evaluate the global spatial clustering of the knowledge scores at 10 incremental distance bands, and the distance band with the highest z-score (highest global clustering) was used for the local cluster analysis [50,51].

#### 5.4.7. Local Spatial Cluster Analysis of Knowledge Scores

The Cluster and Outlier Analysis (Anselin local Moran’s I) Tool was used to identify local clusters of county-level knowledge scores. The “zone of indifference” conceptualization parameter was used to assess the local spatial clustering of the knowledge scores. The zone of indifference conceptualization indicates that all counties within a particular distance band receive the highest weighting, and once this distance is exceeded, the weighting of influence level drops off quickly [50,51]. The distance band with the highest global clustering (124.53 km) identified at the Incremental Spatial Autocorrelation Analysis was used as the critical distance (Figure 6).

## 6. Conclusions

This study demonstrated the utility of combining an online survey with exploratory geographical analysis and spatial statistics to identify global and local clusters of Illinois small animal veterinarians’ knowledge scores regarding AMU and AMR. Areas with higher- and lower-than-expected response rates were also identified, and these areas should be followed up and considered when developing future antimicrobial stewardship programs. The findings of this study can assist animal health stakeholders in designing locally relevant and effective antimicrobial stewardship programs to improve judicious antimicrobial use to limit the emergence of AMR.

## Figures and Tables

**Table 1:** Demographic characteristics of Illinois small animal veterinarians.

Questionnaire Items	Number of Responses	Percentage	95% CI
**Sex**	***n* = 83**		
Female	55	66.3%	55.05, 76.28
Male	26	31.3%	21.59, 42.44
Prefer not to say	2	2.4%	0.29, 8.43
**Age**	***n* = 80**		
50+	39	48.8%	37.41, 60.19
40–49	17	21.2%	12.89, 31.83
30–39	18	22.5%	13.91, 33.21
<30	6	7.5%	2.80, 15.61
**Type of practice facility**	***n* = 83**		
Veterinary Primary Care Hospital/Clinic	62	74.7%	63.96, 83.61
Veterinary Academic Teaching Hospital	12	14.5%	7.70, 23.89
Veterinary Emergency Hospital/Clinic	6	7.2%	2.70, 15.07
Veterinary Referral Hospital	2	2.4%	0.29, 8.43
Veterinary Mobile Practice	1	1.2%	0.03, 6.53
Other	0	0.0%	0.00, 4.35
**Type of clinical practice**	***n* = 83**		
Exclusively small animal (Sum of Canine and Feline is at least 90% of the contact)	63	75.9%	65.27, 84.62
Predominantly small animal (Sum of Canine and Feline is at least 50% of the contact)	10	12.1%	5.93, 21.04
Mixed practice facility (Varied species with at least 25% from small animals and 25% from either food animals or equine).	3	3.6%	0.75, 10.20
Predominantly food animals (Sum of (Bovine, Porcine, Ovine/Caprine, Camelid, Cervid, and Poultry) is at least 50% of the contact).	5	6.0%	1.98, 13.50
Other	2	2.4%	0.29, 8.43
**Number of veterinarians working at the clinic/hospital**	***n* = 82**		
1–2	30	36.6%	26.22, 47.95
3–5	29	35.4%	25.12, 46.70
>5	23	28.0%	18.68, 39.06
**Role in the clinic/hospital**	***n* = 83**		
Owner	36	43.4%	32.53, 54.71
Associate Veterinarian	28	33.7%	23.72, 44.95
Clinical Faculty	8	9.6%	4.25, 18.11
Veterinary Resident/Intern	4	4.8%	1.33, 11.88
Locum/Relief Veterinarian	3	3.6%	0.75, 10.20
Other	4	4.8%	1.33, 11.88
**Additional post-DVM degree or certification**	***n* = 83**		
None	64	77.1%	66.58, 85.62
MS/MPH	10	12.1%	5.93, 21.04
Diplomate	6	7.2%	2.70, 15.07
Diplomate, MS/MPH	1	1.2%	0.03, 6.53
PhD	1	1.2%	0.03, 6.53
Diplomate, MS/MPH, PhD	1	1.2%	0.03, 6.53
**Years of experience prescribing antimicrobials to dogs and/or cats**	***n* = 83**		
11+	61	73.5%	62.66, 82.58
6–10	6	7.2%	2.70, 15.07
1–5	13	15.7%	8.61, 25.29
<1	3	3.6%	0.75, 10.20

**Table 2:** Awareness of antimicrobial resistance and antimicrobial use practices among Illinois small animal veterinarians.

Questionnaire Items	Number of Responses	Percentage	95% CI
**How familiar are you with the topic of antibiotic resistance?**	***n* = 78**		
Very familiar	34	43.6%	32.39, 55.30
Moderately familiar	36	46.2%	34.79, 57.82
Somewhat familiar	8	10.2%	4.53, 19.21
Slightly familiar	0	0.0%	0.00, 4.62
Not at all familiar	0	0.0%	0.00, 4.62
**Are you concerned about antibiotic-resistant bacterial infections when treating your patients?**	***n* = 78**		
Very concerned	26	33.3%	23.06, 44.92
Moderately concerned	42	53.8%	42.18, 65.21
Somewhat concerned	7	9.0%	3.68, 17.62
Slightly concerned	3	3.9%	0.80, 10.83
Not at all concerned	0	0.0%	0.00, 4.62
**Are you aware of the current antimicrobial resistance profiles of major bacterial pathogens (e.g., *E. coli*, *Staphylococcus* sp.) in your area?**	***n* = 78**		
Yes	36	46.2%	34.79, 57.82
No	42	53.8%	42.18, 65.21
**Do you perform cytology before treating suspected bacterial infections?**	***n* = 77**		
Always	8	10.4%	4.59, 19.45
Often	28	36.3%	25.70, 48.12
Sometimes	35	45.5%	34.06, 57.21
Rarely	5	6.5%	2.14, 14.51
Never	1	1.3%	0.03, 7.02
**Do you have antimicrobial prescription guidelines at your facility?**	***n* = 76**		
Yes	20	26.3%	16.87, 37.68
No	56	73.7%	62.32, 83.13
**Have you attended any educational programs on antimicrobial use and antimicrobial resistance after graduation?**	***n* = 75**		
Yes	59	78.7%	67.68, 87.29
No	16	21.3%	12.71, 32.32

**Table 3:** Opinion of Illinois small animal veterinarians on the possibility of different antimicrobial use practices’ impact on the selection of antimicrobial resistance.

Questionnaire Items	Number of Responses	Percentage	95% CI
**Likelihood of the following antimicrobial use practices’ contributions to the development of antimicrobial resistance**			
**Prescription of broad-spectrum antibiotics**	***n* = 78**		
Extremely likely	15	19.2%	11.18, 29.73
Likely	42	53.8%	42.18, 65.21
Neutral	14	18.0%	10.17, 28.28
Unlikely	6	7.7%	2.88, 15.99
Extremely unlikely	1	1.3%	0.03, 6.94
**Inadequate dose or duration of antibiotic treatments**	***n* = 77**		
Extremely likely	45	58.4%	46.64, 69.57
Likely	25	32.5%	22.23, 44.10
Neutral	5	6.5%	2.14, 14.51
Unlikely	2	2.6%	0.32, 9.07
Extremely unlikely	0	0.0%	0.00, 4.68
**Empirical antibiotic therapy without performing culture and susceptibility tests**	***n* = 78**		
Extremely likely	14	18.0%	10.17, 28.28
Likely	43	55.0%	43.44, 66.41
Neutral	14	18.0%	10.17, 28.28
Unlikely	7	9.0%	3.68, 17.62
Extremely unlikely	0	0.0%	0.00, 4.62
**Improper infection prevention practices in veterinary facilities**	***n* = 78**		
Extremely likely	13	16.7%	9.18, 26.81
Likely	28	35.9%	25.34, 47.56
Neutral	26	33.3%	23.06, 44.92
Unlikely	10	12.8%	6.32, 22.32
Extremely unlikely	1	1.3%	0.03, 6.94
**Prescribing antibiotics extra-label**	***n* = 78**		
Extremely likely	7	9.0%	3.68, 17.62
Likely	14	18.0%	10.17, 28.28
Neutral	32	41.0%	30.01, 52.75
Unlikely	25	32.0%	21.93, 43.58
Extremely unlikely	0	0.0%	0.00, 4.62

## Data Availability

The original contributions presented in the study are included in the article/supplementary material. Further inquiries can be directed to the corresponding authors.

## Supplementary Materials

The following supporting information can be downloaded at: https://www.mdpi.com/article/10.3390/antibiotics12030542/s1, Figure S1: Map of the counties in the State of Illinois; Figure S2: Spatial autocorrelation of response rates at different incremental distances; Table S1: The number of responders by county; Table S2: Spatial analysis results of Illinois small animal veterinarians’ survey.
