# Supplementary material for: Knowledge and Attitudes of Small Animal Veterinarians on Antimicrobial Use Practices Impacting the Selection of Antimicrobial Resistance in Dogs and Cats in Illinois, United States: A Spatial Epidemiological Approach
Source: Antibiotics (Basel). 2023 Mar 8;12(3):542. doi: 10.3390/antibiotics12030542 (PMC10044024; doi:10.3390/antibiotics12030542)
Supplement: Supplementary file 1 [file antibiotics-12-00542-s001.zip › antibiotics-2257623-Figures.pdf]

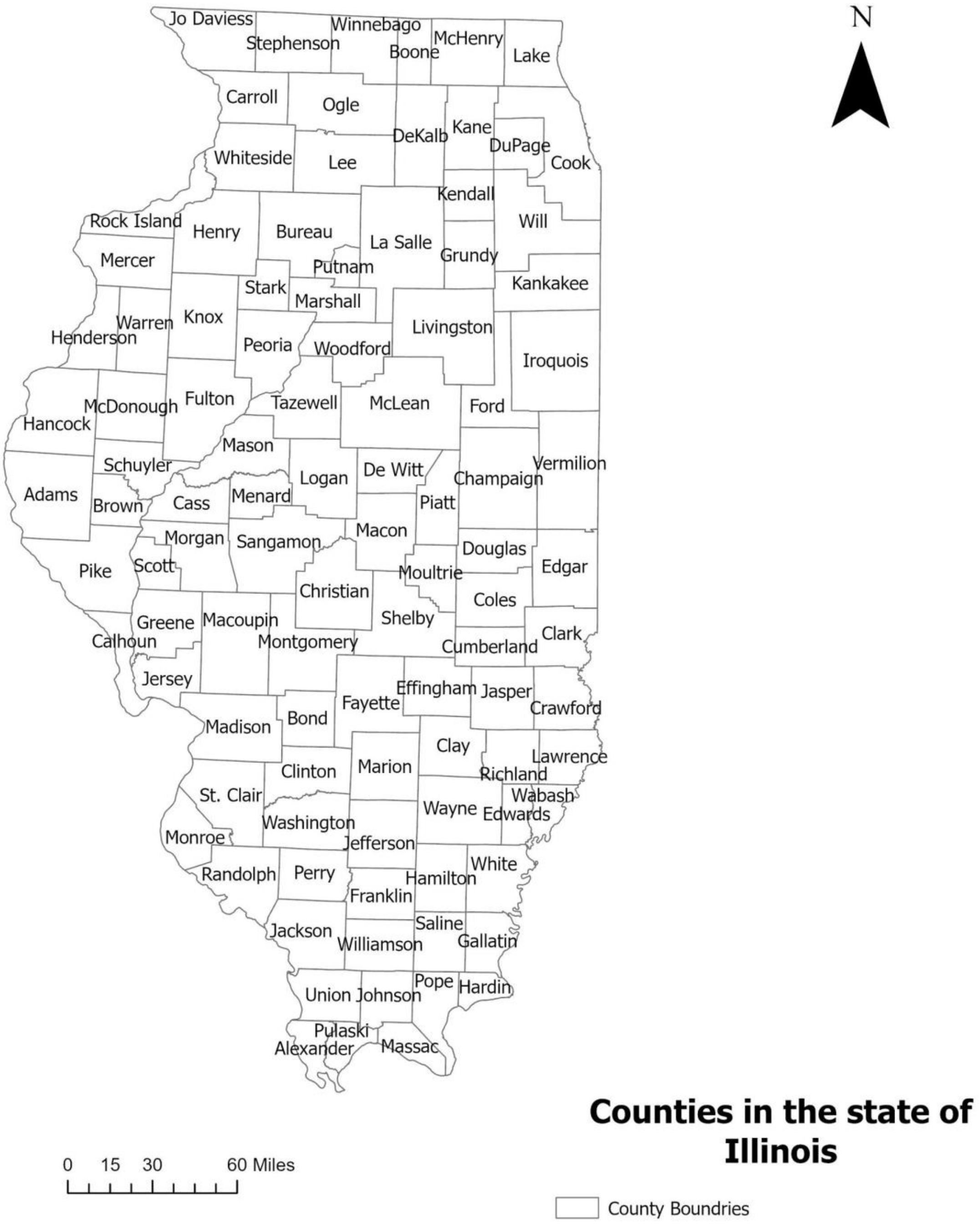

Figuer S1. Map of the counties in the State of Illinois.

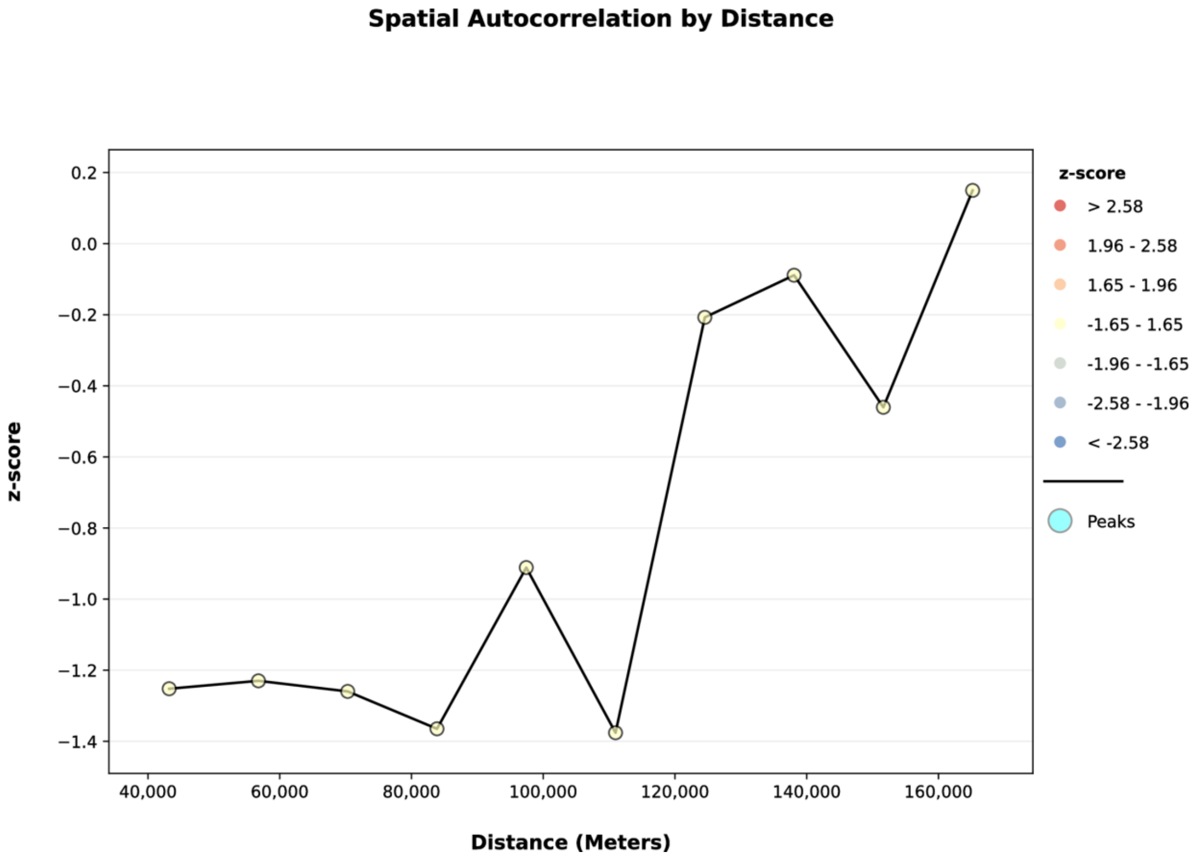

Figure S2. Spatial autocorrelation of response rates at different incremental distances.
