# Supplementary material for: Knowledge and Attitudes of Small Animal Veterinarians on Antimicrobial Use Practices Impacting the Selection of Antimicrobial Resistance in Dogs and Cats in Illinois, United States: A Spatial Epidemiological Approach
Source: Antibiotics (Basel). 2023 Mar 8;12(3):542. doi: 10.3390/antibiotics12030542 (PMC10044024; doi:10.3390/antibiotics12030542)
Supplement: Supplementary file 1 [file antibiotics-12-00542-s001.zip › Table S1.pdf]

**Table S1.** The number of responders by county.

| County name | Responders by county |
|---|---|
| Champaign | 13 |
| Cook | 9 |
| DuPage | 6 |
| Lake | 6 |
| Will | 6 |
| Kane | 4 |
| Carroll | 3 |
| McHenry | 3 |
| Sangamon | 3 |
| Woodford | 3 |
| Adams | 2 |
| Rock Island | 2 |
| Winnebago | 2 |
| Boone | 1 |
| Christian | 1 |
| Coles | 1 |
| Effingham | 1 |
| Fulton | 1 |
| Kankakee | 1 |
| Kendall | 1 |
| Lawrence | 1 |
| Livingston | 1 |
| Macon | 1 |
| McLean | 1 |
| Mercer | 1 |
| Monroe | 1 |
| Moultrie | 1 |
| Peoria | 1 |
| Randolph | 1 |
| St. Clair | 1 |
| Tazewell | 1 |
| Union | 1 |
| Vermilion | 1 |
| Williamson | 1 |
| **Grand Total** | **83** |
