# Supplementary material for: Knowledge and Attitudes of Small Animal Veterinarians on Antimicrobial Use Practices Impacting the Selection of Antimicrobial Resistance in Dogs and Cats in Illinois, United States: A Spatial Epidemiological Approach
Source: Antibiotics (Basel). 2023 Mar 8;12(3):542. doi: 10.3390/antibiotics12030542 (PMC10044024; doi:10.3390/antibiotics12030542)
Supplement: Supplementary file 1 [file antibiotics-12-00542-s001.zip › Table S2.pdf]

Table S2. Spatial analysis results of Illinois small animal veterinarians survey

Analysis	Method	Type of Cluster	County
**Response rate**	Anselin local Moran's I	High-High	-
		High-Low	Carroll, Adams, Lawrence, Effingham
		Low-Low	-
		Low-High	-
**Knowledge score**	Anselin local Moran's I	High-High	Cook, DuPage, Kankakee, Livingston
		High-Low	-
		Low-Low	Hamilton, Wayne, Edwards, Clay, Richland, Lawrence
		Low-High	Iroquois, Ford, La Salle, Putnam
